# Dynamics of Paraspeckle Components in Herpes Simplex Virus 1 (HSV-1)-Infected Human Neuronal Cells

**DOI:** 10.3390/v18050552

**Published:** 2026-05-12

**Authors:** Carolina Filipponi, David C. Bloom, Carlo Gambotto, Callen T. Wallace, Jadranka Milosevic, Simon C. Watkins, Shane Buckley, Maribeth A. Wesesky, Vishwajit L. Nimgaonkar, Leonardo D’Aiuto

**Affiliations:** 1 Department of Psychiatry, University of Pittsburgh School of Medicine, Western Psychiatric Institute and Clinic, 3811 O’Hara Street, Pittsburgh, PA 15213, USA; carolina.filipponi@phd.unipi.it (C.F.); weseskyma@upmc.edu (M.A.W.); vishwajitnl@upmc.edu (V.L.N.); 2 Department of Translational Research and New Technologies in Medicine and Surgery, Retrovirus Center, University of Pisa, 56126 Pisa, Italy; 3 Department of Molecular Genetics & Microbiology, University of Florida College of Medicine, Gainesville, FL 32611, USA; dbloom@ufl.edu; 4 Department of Biological Sciences, Dietrich School of Arts and Science, University of Pittsburgh, 4249 Fifth Avenue, Pittsburgh, PA 15260, USA; cag339@pitt.edu; 5 Department of Cell Biology, University of Pittsburgh, 3500 Terrace Street, S362 Biomedical Science Tower (South), Pittsburgh, PA 15261, USA; callenwall@gmail.com (C.T.W.); simon.watkins@pitt.edu (S.C.W.); 6 Captis Diagnostics, Pittsburgh, PA 15213, USA; jmilosevic@captisdx.com; 7 Department of Human Genetics, School of Public Health, University of Pittsburgh, 130 De Soto St., Pittsburgh, PA 15261, USA; tshanebuckley@gmail.com

**Keywords:** Herpes Simplex Virus 1 (HSV-1), paraspeckles, human induced pluripotent stem cells (hiPSCs), neural precursor cells (NPCs), neurons, brain organoids

## Abstract

Paraspeckles are subnuclear ribonucleoprotein condensates that regulate host stress responses, including those triggered by viral infection. In vitro studies using non-neuronal cells have shown the involvement of specific paraspeckle components in facilitating the replication of certain viruses, including Herpes Simplex Virus 1 (HSV-1), but these processes have not been investigated in human neuronal cells, which represent a relevant target of the virus. We employed human neural precursor cells (NPCs), neurons, and brain organoids derived from hiPSCs to investigate the previously unexplored dynamics of paraspeckle components in HSV-1-infected human neuronal cells. Our results reveal cell-type-specific differences in the expression of paraspeckle genes in response to HSV-1 infection. Unlike other viruses, HSV-1 orchestrates a previously unreported redistribution of paraspeckle proteins, leading to their accumulation in viral replication compartments (VRCs). Importantly, the expression of the paraspeckle proteins NONO and SFPQ correlates with HSV-1 permissiveness in human neuronal cells and may be required to establish a nuclear environment favoring viral transcription/replication. This enhances our understanding of how stress-response pathways in cells can be exploited by viruses in a cell-type-specific manner.

## 1. Introduction

Paraspeckles are small membraneless subnuclear ribonucleoprotein bodies located in the interchromatin space, which play crucial roles in regulating gene expression by several mechanisms, such as retention of specific RNAs in the nucleus [1], sequestration of proteins [2], and binding to gene promoters (thus, influencing their expression) [3]. Paraspeckles are assembled through interactions between the long non-coding RNA NEAT1 (Nuclear Enriched Abundant Transcript 1) [4,5] and specific RNA-binding proteins, primarily members of the DBHS (Drosophila Behavior Human Splicing) family, including PSF/SFPQ, P54NRB/NONO, and PSPC1 (Paraspeckle Protein 1) [5,6]. Mouse CNS cells generally have fewer paraspeckles than their human counterparts [7], suggesting that there are species-specific or cell-specific variations. Paraspeckles are indispensable for host stress responses, such as those during viral infection, functioning as global sensors of cellular stress and contributing to antiviral defense mechanisms [4,8,9]. Their dynamic nature allows them to respond to cellular needs and contributes to the fine-tuning of gene expression in different contexts. For example, Kaposi’s sarcoma-associated herpesvirus (KSHV) infection induces the formation of structurally distinct paraspeckles characterized by increased size and altered protein composition [9]. These modified paraspeckles are essential for productive lytic replication, potentially serving as RNA processing hubs that facilitate viral transcript maturation during infection [9]. Similarly, Epstein–Barr virus (EBV) utilizes paraspeckle proteins to modulate its lytic cycle through interaction of its EBER2 RNA with paraspeckle proteins, such as SFPQ, NONO, and RBM14 [10].

HSV-1 infection of HeLa cells and mouse embryonic fibroblasts (MEFs) [11] rapidly activate STAT3, leading to a STAT3-dependent upregulation of NEAT1 expression. This increase in NEAT1 enhances paraspeckle formation, coinciding with a redistribution of both nuclear speckles and paraspeckles within infected nuclei. Concurrently, the nuclear speckle protein SRSF2 interacts with paraspeckle components, including NEAT1, PSPC1, and NONO. These factors, along with SRSF2, bind HSV-1 genomic DNA [11]. PSPC1 plays a crucial role in recruiting STAT3 to paraspeckles, facilitating STAT3’s interaction with viral gene promoters, including those encoding ICP0 and thymidine kinase (TK). In contrast, SFPQ is a negative regulator of HSV-1 replication. Specifically, knockdown of SFPQ leads to increased transcription of key viral genes, such as ICP0 and TK, indicating that SFPQ plays a crucial role in suppressing viral gene expression during infection. Furthermore, NEAT1 and SRSF2 associate with epigenetic modifiers, such as EZH2 and the P300/CBP complex, promoting histone modifications near viral genes [3]. This coordinated action of speckle and paraspeckle components, involving both direct DNA binding and epigenetic modulation, ultimately enhances viral gene transcription and replication, illustrating a sophisticated mechanism by which HSV-1 exploits host nuclear structures to promote its life cycle.

However, the dynamics of paraspeckle proteins in HSV-1-infected neuronal cells have yet to be investigated. In this study, we investigated, for the first time, the effect of HSV-1 infection on the gene expression of key paraspeckle components and analyzed their expression dynamics in human neural precursor cells (NPCs), neurons, and brain organoids derived from human induced pluripotent stem cells (hiPSCs).

Our results show cell-type-specific differences in paraspeckle gene expression between NPCs and neurons. Key paraspeckle proteins redistribute in infected cells, accumulating in VRCs. In infected neuronal cells, the viral protein ICP8 was mainly observed in cells with detectable levels of NONO and SFPQ, suggesting that these proteins correlate with HSV-1 permissiveness in human neuronal cells.

Our results reveal previously unrecognized paraspeckle dynamics in HSV-1-infected neurons, thereby providing an important foundation for future mechanistic studies.

## 2. Materials and Methods

### 2.1. Cell Lines

The hiPSC lines SC0000019 (subclone SF) and SC0000020 (subclone SD) (RUCDR Infinite Biologics, Piscataway, NJ, United States) were employed to generate monolayer cultures of neurons and brain organoids, respectively. The hiPSCs were established at the National Institute of Mental Health (NIMH) Center for Collaborative Studies of Mental Disorders—funded by Rutgers University’s Cell and DNA Repository (RUCDR) (Subject (Lab) Code: 73-56010-01 and 73-5610-02; University of Pittsburgh ID: O1-SD and O2-SF; Line type: iPSC; RUCDR IDs: SC0000019 and SC0000020; Rutgers Clone Aliases: R139361416_SD; R149384581_SD and R139361417_SF; R149575079_SF; Subclone: SD and SF; Source cells: fibroblasts (both); Reprogramming Method: Sendai (both)). The control steps included analysis of the pluripotency markers NANOG, Oct4, TRA-1-60, TRA-181, SOX2 and SSEA4. We subsequently conducted karyotyping, array comparative genomic hybridization (aCGH) assays and short tandem repeat (STR) profiling and compared them with donor genomic DNA to evaluate structural changes in genomic DNA during the generation of hiPSC lines. Cultures were periodically tested for mycoplasma contamination.

### 2.2. Virus Preparation

The HSV-1 strain 17*syn*^+^ (gift from Dr. D. Bloom from stocks obtained from Dr. J. Stevens) was employed in this study. The virus stock was prepared in the D’Aiuto laboratory at the University of Pittsburgh. A total of 80–90% confluent monolayers of Vero (ATCC, Manassas, VA, USA) cells were infected at a multiplicity of infection (MOI of 3) in DMEM (ThermoFisher, Waltham, MA, USA) medium supplemented with 2% FBS (ThermoFisher, Waltham, MA, USA). After 2 h, the inoculum was removed, and cells were washed and cultured for 2–3 days, until the appearance of full cytopathic effect (CPE). The cells were scraped and transferred along with the culture supernatant into 15 mL conical tubes. Cells were centrifuged at 1000 rpm for 5 min. The culture supernatant was removed, leaving behind 1.5 mL, and the cell pellet was resuspended using a vortex for 1–2 min. Cells were freeze-thawed three times. Debris was then removed by centrifuging at 3000 rpm for 5 min, and the top culture supernatant containing cell-free viral particles was stored at −80 °C until use. Virus titers were determined by standard plaque assay [12].

### 2.3. Generation of Monolayer Cultures of hiPSC-Derived NPCs and Neurons

Neuronal precursor cells (NPCs) were derived from hiPSCs as follows. hiPSCs were seeded at a density of 10^6^ cells/well in 6-well plates coated in Matrigel (Corning Life Sciences, Tewksbury, MA, USA). Cells were cultured in mTeSR1 supplemented with the dual SMAD inhibitors SB431542 (10 μM) (MilliporeSigma S4317-5MG, Burlington, MA, USA) and LDN193189 (100 nM) (MilliporeSigma SML055-9-25MG, Burlington, MA, USA) and Rho-associated protein kinase inhibitor (ROCK inhibitor) Y27632 (StemCell Technologies, Vancouver, BC, Canada) (10 mM) for 5–7 days until the appearance of neural rosettes. The ROCK inhibitor was withdrawn after 24 h. Cells were then dissociated with Accutase^TM^ (StemCell Technologies, Vancouver, BC, Canada) and seeded at a density of 10^6^ cells/well in Matrigel-coated 6-well plates and cultured in mTeSR1 Plus supplemented with dual SMAD inhibitors and a ROCK inhibitor. After 5–7 days NPCs were dissociated and expanded in the StemDiff^TM^ Neural progenitor medium (StemCell Technologies, Vancouver, BC, Canada) in Matrigel-coated 6-well plates (10^6^ cells/well).

NPCs were seeded into Matrigel-coated 12- or 6-well plates to a density of 2.5 × 10^5^ or 5 × 10^5^ cells/well, respectively, and cultured in the neurobasal medium [neurobasal medium supplemented with 0.5X B27 (Vitamin A+), 1X penicillin–streptomycin (P/S), 1X Glutamax, BDNF (Cambridge, MA, USA) (10 ng/mL), CHIR99021 (BioGems, Inc., Westlake Village, CA, USA) (3 µM), Dorsomorphin (BioGems, Inc., Westlake Village, CA, USA) (1 µM), Forskolin (Tocris Bioscience, Minneapolis, MN, USA), and a ROCK inhibitor (10 mM)]. Two days later, CHIR99021, Dorsomorphin, Forskolin (10 µM), and the ROCK inhibitor were withdrawn, and differentiating NPCs were cultured for 4 weeks. The half medium was changed every other day.

### 2.4. Infection of Monolayer Cultures of hiPSC-Derived NPCs and Neurons

A cell-free virus was adsorbed onto monolayer hiPSC-derived NPCs and neurons at a multiplicity of infection (MOI) of 0.1 and 1, respectively. One hour after the infection, the inocula were then removed, and the cells were washed twice with Dulbecco’s modified eagle medium (DMEM)–Ham’s F-12 medium, and NPCs and neurons were cultured for 24 h in the StemDiff^TM^ Neural progenitor medium and neurobasal medium, respectively.

### 2.5. Generation of Brain Organoids

Organoids were generated as previously described [13] with some modifications. Briefly, hiPSCs cultured with the mTeSR^TM^ plus medium (StemCell Technologies, Vancouver, BC, Canada) in Matrigel-coated 6-well plates were detached with Accutase and then dissociated into single-cell suspension by gently pipetting. Cells were resuspended in the mTeSR^TM^ plus medium supplemented with the ROCK inhibitor (StemCell Technologies, Vancouver, BC, Canada) (10 mM) and rhFGF-basic (20 ng/mL) and seeded into low attachment 96-well plates (Nunclon Sphera 3D culture system, ThermoFisher, Waltham, MA, USA) at a density of 10,000 cells/well to allow for the formation of embryoid bodies. On day 3, the medium was replaced with the Essential-6 medium (Gibco, A1516401, Waltham, MA, USA) supplemented with the dual SMAD inhibitors SB431542 (10 µM) (MilliporeSigma S4317-5MG, Burlington, MA, USA) and LDN193189 (100 nM) (MilliporeSigma SML055-9-25MG, Burlington, MA, USA). The half medium was changed every other day. On day 8, the medium was replaced with the Neuronal medium [DMEM/F12 supplemented with a 1X MEM Nonessential Amino Acid supplement (MEM-NEAA, CORNING 25-025-CI, Corning, NY, USA), 1X Glutamax (Gibco 35050-061, Waltham, MA, USA), 1X an N2 supplement (Gibco 17502-048, Waltham, MA, USA), 1 µg/mL of Heparin (StemCell Technologies 07980, Vancouver, BC, Canada), 20 ng/mL of rhFGF-basic, and 20 ng/mL of rhEGF] in untreated 10 cm petri dishes (Corning, Cat# 430591, Corning, NY, USA). The half medium was changed every other day. On day 16, spheres were transferred into 10 cm petri dishes in the Neuronal medium and placed on an orbital shaker in an incubator at 70 rpm (Orbi-Blotter^TM^, BioLink Laboratories LLC, Washington, DC, USA). The medium was changed every 3 days. On day 20, the culture medium was changed to cortical organoid differentiation medium I [CODM-I: DMEM:F12/Neurobasal (1:1 *v*/*v*) supplemented with 1X Glutamax, 1X B-27 (VitA[−]), 0.5X Non-essential amino acids, 0.5X N-2, Insulin (2.5 mg), 1X penicillin–streptomycin (P/S), 20 ng/mL of rhFGF-basic, and 20 ng/mL of rhEGF]. Five days later, the CODM-I medium was replaced with cortical organoid differentiation medium II (CODM-II: DMEM/F12–Neurobasal (1:1 *v*/*v*) supplemented with 1X Glutamax, 1X B-27 (VitA[+]), 0.5X Non-essential amino acids, 0.5X N-2, Insulin (2.5 mg), BDNF (10 ng/mL), and 1X P/S). The culture medium was changed every other day. On day 42, the CODM-II medium was replaced with the BrainPhys^TM^ Neuronal medium (StemCell Technologies, Vancouver, BC, Canada).

### 2.6. Infection of Brain Organoids

Sixty-day-old brains were transferred singularly in 1.5 mL Eppendorf tubes and washed with 500 μL of the DMEM-F12 medium. The medium was then discarded, and 50 μL of the neurobasal medium containing 3000 PFU of HSV-1, strain 17*syn*^+^, was added. After perforating the cap of Eppendorf tubes using a 20-gauge sterile needle, the organoids were cultured in an incubator under standard conditions (5% CO_2_, 37 °C, and 100% humidity). After 2 h, the inocula were removed, and the organoids were washed and cultured in the BrainPhys^TM^ Neuronal medium (StemCell Technologies, Vancouver, BC, Canada). Uninfected and infected organoids were processed for immunohistochemistry on day 3 post-infection.

Two independent batches of organoids were generated from the human iPSC lines SC0000019 and SC0000020, with 8–10 organoids per condition analyzed for each iPSC line. Quantitative analyses were performed on three organoids per condition.

### 2.7. Western Blotting

Protein lysates from uninfected and infected neuronal cultures were prepared using the RIPA Lysis Buffer (Thermo Fischer Scientific, Waltham, MA, USA). Protein concentrations were determined using the BCA Protein Assay Kit (Thermo Fischer Scientific, Waltham, MA, USA). Equal amounts of total protein (20 μg) were mixed with 4× the Laemmli buffer. Samples were heated at 70 °C for 10 min. Cell lysates were separated by electrophoresis on SDS–polyacrylamide gels and transferred onto nitrocellulose membranes using the iBlot^TM^ 3 Western Blot Transfer System (Invitrogen^TM^, Thermo Fisher Scientific, Waltham, MA, USA). Membranes were blocked for 1 h at room temperature in 5% nonfat dry milk in PBS Tween 0.1%. After blocking, the membranes were incubated overnight at 4 °C with rabbit anti-SFPQ (Abcam, Cambridge, UK, Cat# ab177149, dilution 1:1000), rabbit anti-NONO (Abcam, Cambridge, UK, Cat# ab133574, dilution 1:1000), rabbit anti-PSPC1 (Abcam, Cambridge, UK, Cat# ab104238, dilution 1:1000), and mouse anti-GAPDH (Santa Cruz Biotechnology, Dallas, TX, USA, Cat #sc-365062, dilution 1:2000). After washings, the blots were incubated with HPRO-conjugated anti-mouse IgG (Abcam, Cambridge, UK, Cat# ab6789, dilution 1:2000) and anti-rabbit IgG (Abcam, Cambridge, UK, Cat# ab6721, dilution 1:2000) for 1 h at room temperature and imaged with iBright 750 (Invitrogen, Thermo Fisher Scientific, Waltham, MA, USA). Protein bands were detected using enhanced chemiluminescence (ECL) detection (SuperSignal^TM^ West Pico PLUS Chemiluminescent Substrate, Thermo Fisher Scientific, Waltham, MA, USA) according to the manufacturer’s recommendations. Exposure times were optimized to maintain signals within the linear range of detection and to prevent saturation. Images were captured using iBright 750 (Invitrogen). Band intensities were quantified by densitometry using Fiji (ImageJ, v1.54p). However, the GAPDH loading control showed some variability across lanes, and the data should therefore be interpreted as semiquantitative.

### 2.8. RNA Isolation, Reverse Transcription, and Quantitative PCR

Total RNA was extracted from uninfected or HSV-1-infected hiPSC-derived NPCs and neurons using the Qiagen RNeasy Mini Kit, following the manufacturer’s protocol. RNA quantity and purity were assessed by Qubit 4 (Thermo Fisher Scientific, Waltham, MA, USA). For cDNA synthesis, 200 ng of total RNA was reverse-transcribed using the ProtoScript II First Strand cDNA Synthesis Kit (New England Biolabs, Ipswich, MA, USA) together with the supplied random primer mix. Reactions were performed according to the manufacturer’s instructions to generate first-strand cDNA suitable for quantitative analysis.

RT-qPCR was carried out using the LUNA Universal Probe qPCR Master Mix (New England Biolabs). The following primers were used: NEAT1_2 Fw: 5′-CGGAGGGTCTTGTAACACCAG-3′; NEAT1_2 Rev 5′-AGTCCGGGCAACACAGAAAG-3′; NONO Fw: 5-TGATGAAGAGGGACTTCCAGA-3′; NONO Rev: 5′-AGCGCATGGCATATTCATACT-3′; SFPQ Fw: 5′-ACAGGGAAAGGCATTGTTGA-3′; SFPQ Rev: 5′-TCATCTAGTTGTTCAAGTGGTTCC-3′; PSPC1 Fw: 5′-GGGCGAGAAGACGTACACG-3′; PSPC1 Rev: 5′-AAGCGAATCCGTAGAGGTCTG-3′; HPRT Fw: 5′-CATTATGCTGAGGATTTGGAAAGG-3′; and HPRT Rev: 5′-CTTGAGCACACAGAGGGCTACA-3′.

Reactions were run on a CFX-OPUS 96 real-time PCR thermocycler (Bio-Rad, Hercules, CA, USA). Relative transcript levels were quantified using the ΔΔCt method and normalized to HPRT as the endogenous control.

### 2.9. Immunofluorescence

The two-dimensional (2D) neuronal cultures were fixed with 4% paraformaldehyde (PFA) and permeabilized with 0.2% Triton-X before immunostaining. Frozen sections of organoids were prepared as follows. Organoids were fixed in 4% paraformaldehyde (PFA) for 4 h, washed in PBS, and cryoprotected in 20% sucrose until they sank (approximately 24 h). Organoids were then transferred into cryomolds (Tissue-Tek Cryo Mold Intermediate, Sakura Finetek USA, Inc., Torrance, CA, USA). After adsorbing traces of the culture medium, the organoids were embedded into the OCT medium in cryomolds (Tissue-Tek Cryo Mold Intermediate, Sakura Finetek USA, Inc., Torrance, CA, USA) and frozen at −22 °C. Sections of 10 µm were prepared by Cryostat (Micron HM350; Thermo Fisher Scientific, Waltham, MA, USA). From each condition, a minimum of 30 sections were obtained by cryosectioning the organoids. Each cryosection contained 2–3 organoids. At least 3 slides from each condition have been analyzed. To prevent sampling the same cells only sections that were 50 mm apart were used. Frozen sections were stored at −80 °C until needed. Before staining, frozen sections were fixed with 4% PFA for 20 min and incubated with 10% Goat serum (Thermo Fisher Scientific, Waltham, MA, USA, Cat# 50062Z) and 0.2%Triton-X 100 (Millipore Sigma/Sigma-Aldrich, St. Louis, MO, USA, Cat# X-100) for 1 h at room temperature.

Samples were incubated with primary antibodies overnight at 4 °C. Primary antibodies used were mouse monoclonal anti-HSV-1 ICP8 (Abcam, Cambridge, UK, Cat# ab20194, dilution 1:500), rabbit polyclonal anti-p54nrb (Abcam, Cambridge, UK, Cat# ab133574, dilution 1:250), rabbit polyclonal anti-SFPQ (Abcam, Cambridge, UK, Cat# ab177149, dilution 1:250) and rabbit polyclonal anti-PSPC1 (Abcam, Cambridge, UK, Cat# ab104238, dilution 1:250).

The following fluorophore-conjugated secondary antibodies were used to detect bound primary antibodies: Alexa Fluor 488 goat anti-rabbit (Thermo Fisher Scientific, Waltham, MA, USA, Cat# 1:300 dilution), Alexa Fluor 488 goat anti-mouse (Thermo Fisher Scientific, Waltham, MA, USA, Cat# A-10680, 1:300 dilution), Alexa Fluor 594 goat anti-rabbit (Thermo Fisher Scientific, Waltham, MA, USA, Cat# A-11012, 1:300 dilution), and Alexa Fluor 594 goat anti-mouse secondary antibody (Thermo Fisher Scientific, Waltham, MA, USA, Cat# A-11005, 1:300 dilution). Images were acquired using a Nikon Ti inverted microscope and Nikon A1 point scanning confocal scan head.

Pearson’s R coefficient and Manders’ overlap coefficient to quantify colocalization between ICP8 and NONO, SFPQ, or PSPC1 in HSV-1-infected monolayer cultures of neurons were calculated using the JaCoP plug-in feature in Fiji (ImageJ, v1.54p), an open-source platform based on ImageJ for biological-image processing [14]. A threshold of R > 0.5 was set to define high colocalization between the two markers, as this value is generally considered to represent a strong positive correlation. Statistical analysis was performed using the R programming language. The ‘aov’ function was used to calculate the initial ANOVA across hpi. Following the initial ANOVA, a Tukey Honest Significant Difference test was performed from each time point against the initial 6 hpi using the ‘TukeyHSD’ function. Boxplot graphs were generated within R (version 4.5.1) as well by using the ggplot library.

Confocal z-stacks of immunostained brain organoids were acquired with a 60× 1.40 N.A. objective at 250 nm steps through the entire volume of the cell. Image analysis was performed using Nikon’s NIS Elements analysis software (Version 6.10.02). Image processing was performed using NIS Elements AI Denoising, and the positive signal was segmented using an intensity-based threshold. Object-based colocalization was performed on confocal z-stacks by measuring positive voxels for both NONO (red) and ICP8 (green) signals. The resulting data reflect the area of the positive NONO or ICP8 signal alone as well as colocalized regions positive for the simultaneous expression of both NONO and ICP8.

The fluorescence intensity of nuclear NONO, SFPQ, and PSPC1 in uninfected and infected organoids was analyzed by determining the corrected total cell fluorescence (CTCF) using Fiji (ImageJ, v1.54p) and normalized for background readings using the following equation: CTCF = Integrated Density − (Area of selected cell × Mean fluorescence of background readings).

## 3. Results

### 3.1. Dynamics of Paraspeckles in HSV-1-Infected Monolayer Neuronal Cultures

**Figure 1:**
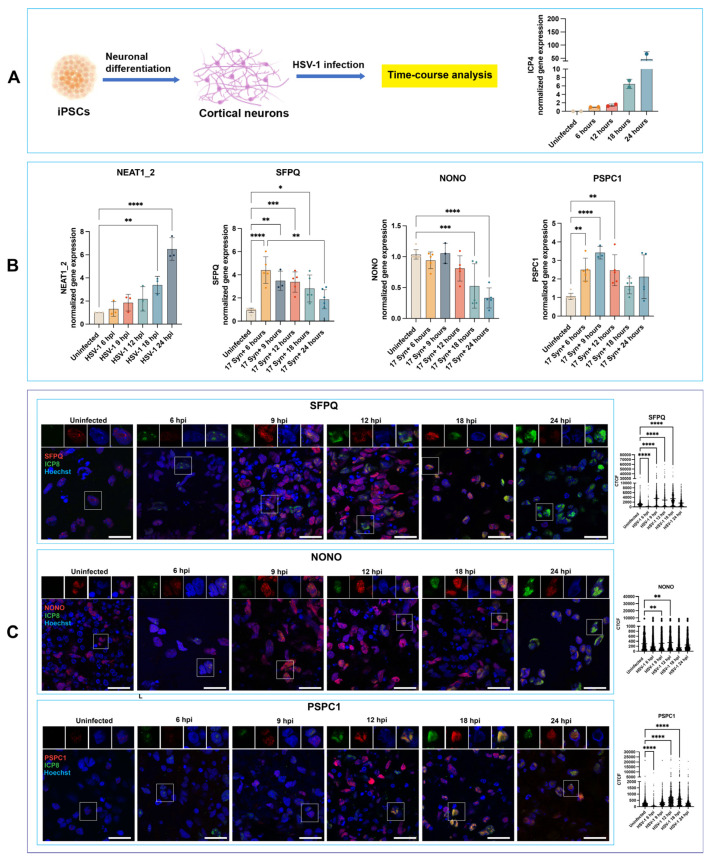
**Temporal changes in paraspeckle components NEAT1_2, NONO, SFPQ, and PSPC1 in response to HSV-1 infection in human neurons.** Four-week-old hiPSC-derived human neuronal cultures were infected with HSV-1, strain 17*syn*^+^, at an MOI of 1 (**A**). The expression of the immediate early HSV-1 gene ICP4 (**A**) and paraspeckle genes NEAT1_2, NONO, SFPQ, and PSPC1 (**B**) were analyzed at the specified time points post-infection using RT-qPCR. The redistribution of NONO, SFPQ, and PSPC1 proteins in infected nuclei was analyzed by immunocytochemistry (ICC) (**C**). RT-qPCR analysis showed NEAT1_2 progressively upregulated in infected neurons, achieving statistical significance at 18 hpi; NONO expression remained stable initially but was significantly down-regulated at 18 h; PSPC1 expression increased progressively, peaking at 12 hpi, followed by a decrease to baseline levels; and SFPQ showed a significant increase at 6 hpi, maintaining elevated levels despite partial decline (**B**). ICC analysis showed that these paraspeckle proteins redistribute to Hoechst-negative, ICP8-positive nuclear regions, likely corresponding to VRCs. The fluorescence intensities of SFPQ and PSPC1, analyzed using the Corrected Total Cell Fluorescence (CTCF) method, transiently increased during HSV-1 infection, peaking between 9 and 18 h post-infection and returning to the baseline by 24 h, while NONO showed a similar peak between 9 and 12 h but returned to the baseline earlier, by 18 h, indicating that their primary functional roles occur during the early to mid-stages of infection (**C**). RT-qPCR data are normalized to GAPDH. One-way analysis of variance (ANOVA), followed by Tukey’s post hoc multiple comparison test, was used to compare gene expression levels (**B**) and CTCF (**C**) between uninfected and infected conditions. RT-qPCR: **** *p* < 0.0001, *** *p*  <  0.001, and ** *p* = 0.0099 for temporal expression of NEAT1_2 and *p*  <  0.01 for temporal expression of PSPC1 and SFPQ; CTCF: **** *p*< 0.0001, ** *p*  <  0.01, and * *p* < 0.05. Nuclei were counterstained with Hoechst 33342 (blue). Scale bars: 25 μm.

To date, the consequences of HSV-1 infection on paraspeckles have been investigated in HeLa cells, MEFs, and C-33A cells [3,11] but remain unexplored in neuronal cells, which represent the reservoir of latent infection and the source of virus for recurrent disease. To this end, we focused on hiPSC-derived neuronal cultures and conducted a time-course analysis to elucidate the temporal changes in paraspeckle gene expression during HSV-1 infection in a mature neuronal model (Figure 1A). NPCs were generated from hiPSCs and differentiated into neurons as previously described [15]. After 4 weeks of differentiation, neurons were infected with HSV-1, strain 17*syn*^+^, at a multiplicity of infection (MOI) of 1 and subsequently processed for RT-qPCR, Western blotting, and immunofluorescence analyses at 6, 9, 12, 18, and 24 h post-infection (hpi).

RT-qPCR analysis showed a progressive increase in the expression of the NEAT1_2 isoform (which acts as a crucial structural scaffold for paraspeckle assembly [16]), reaching statistical significance starting from 18 hpi (*p* = 0.0099) onward (Figure 1B). This expression profile differs from that reported in infected HeLa cells and MEFs [11], where NEAT1_2 is transiently upregulated early in infection, peaking at around 4 hpi, then declining but remaining above the levels observed in uninfected controls. The expression of the paraspeckle gene NONO in infected neurons exhibited a significant decrease starting at 18 hpi (*p* = 0.0008), while the expression of PSPC1 showed a significant increase at 6, 9, and 12 hpi (*p* = 0.0037, *p* < 0.0001, and *p* = 0.0047), followed by a reduction to levels comparable to those in uninfected cultures at later time points (Figure 1B). A significant increase in SFPQ expression was detected at 6 hpi (*p* < 0.0001), followed by a decrease at later time points; however, SFPQ transcript levels remained significantly higher than those observed in uninfected cultures across the subsequent time points (Figure 1B). The transcription level of the immediate early HSV-1 gene ICP4 at the different time points is shown in Figure 1A.

Immunocytochemistry analysis of HSV-1 -infected neurons showed that SFPQ and NONO undergo nuclear redistribution, accumulating in Hoechst-negative nuclear regions that are positive for ICP8, which likely represent viral replication compartments (VRCs; Figure 1C). Quantitative colocalization analysis further substantiated this redistribution, demonstrating that both NONO and SFPQ progressively reorganize during HSV-1 infection and increasingly associate with ICP8-defined VRCs. Specifically, Pearson’s correlation coefficients and overlap coefficients increased significantly across post-infection time points for both proteins (NONO 6 → 24 hpi *p* < 0.001, SFPQ 6 → 24 hpi *p* < 0.001), indicating that the association of NONO and SFPQ with ICP8 became more robust as infection progressed. Additional colocalization analyses provided further details of these redistributions. Manders’ M2 fractions, which quantify the proportion of the NONO or SFPQ signal present within ICP8-positive regions, increased significantly from early to late infection for both proteins, with several pairwise comparisons remaining significant after Tukey’s multiple pairwise correction (e.g., NONO 6 → 24 hpi *p* < 0.001, SFPQ 6 → 24 hpi *p* < 0.001 (Figure S1).

A similar analysis of PSPC1 revealed that this paraspeckle-associated protein also exhibits time-dependent recruitment toward ICP8-positive replication structures, although with a lower magnitude compared to SFPQ and NONO. A significant increase in Pearson’s correlation (*p* ≈ 7.6 × 10^−4^ Tukey-adjusted), overlap coefficients (*p* ≈ 3.1 × 10^−2^ Tukey-adjusted) and Mander’s M2 values (*p* ≈ 6.1 × 10^−4^ Tukey-adjusted) was observed between 6 and 24 hpi, indicating that PSPC1 accumulates into ICP8-positive viral replication compartments.

Together, these results suggest coordinated redistribution of specific paraspeckle proteins to VRCs during HSV-1 infection.

The fluorescence intensity of SFPQ and PSPC1 increased significantly at 9 hpi and 12 hpi (*p* < 0.0001), respectively, but decreased to a level comparable to uninfected cells at 24 hpi (Figure 1C). A significant increase in NONO fluorescence intensity was observed between 9 hpi and 12 hpi, followed by a decrease at later time points (Figure 1C). This temporal pattern indicates that SFPQ, NONO, and PSPC1 are dynamically regulated during HSV-1 infection, with their nuclear abundance peaking during 9–18 hpi, possibly to facilitate viral gene expression or replication. The reduction in SFPQ, NONO, and PSPC1 fluorescence intensity at 24 hpi (Figure 1C) suggests that their roles are most critical during the early to mid-stages of infection, after which their involvement diminishes as the infection progresses.

**Figure 2:**
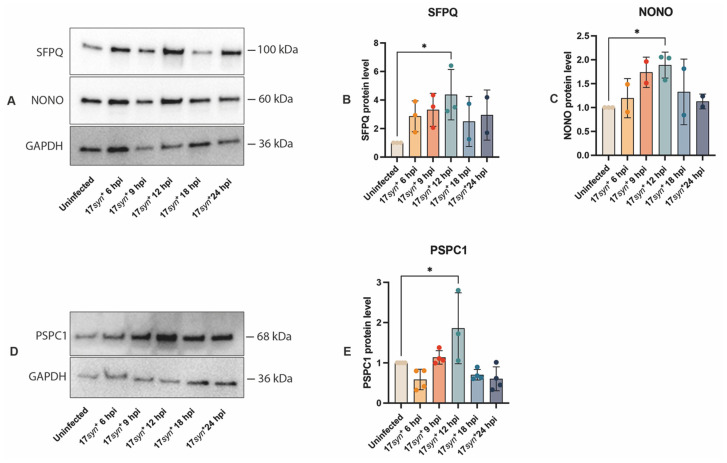
**Western blotting of temporal expression of SFPQ, NONO, and PSPC1 during HSV-1 infection of hiPSC-derived neurons.** (**A**) Representative Western blot showing protein expression levels of SFPQ and NONO at the indicated time points post-infection. GAPDH was used as a loading control for normalization. (**B**,**C**) Densitometric quantification of SFPQ and NONO protein levels, normalized to GAPDH and expressed relative to the uninfected control. (**D**) Representative Western blot showing protein expression levels of PSPC1 at the indicated time points post-infection. GAPDH served as a loading control. (**E**) Quantitative analysis of PSPC1 protein expression normalized to GAPDH and expressed relative to the uninfected control. Quantitative analyses were performed on samples derived from three independent neuronal cultures per condition. Data represent the mean ± SD from three independent experiments. Statistical significance was determined using one-way ANOVA, followed by Tukey’s post hoc test, with *p* < 0.05 (*) considered statistically significant.

Western 1 increases transiently in HSV-1-infected neurons, peaking at 12 hpi (*p* = 0.0409, *p* = 0.0465, and *p* = 0.0230) and subsequently declines to levels comparable to those observed in uninfected cells. This temporal pattern supports the idea that these proteins are specifically recruited or required during initial viral gene expression and nuclear replication events. However, the variability in the GAPDH loading control suggests that these results should be interpreted as semiquantitative rather than strictly quantitative (Figure 2 and Figure S2).

### 3.2. Dynamics of Paraspeckles in HSV-1-Infected Brain Organoids

**Figure 3:**
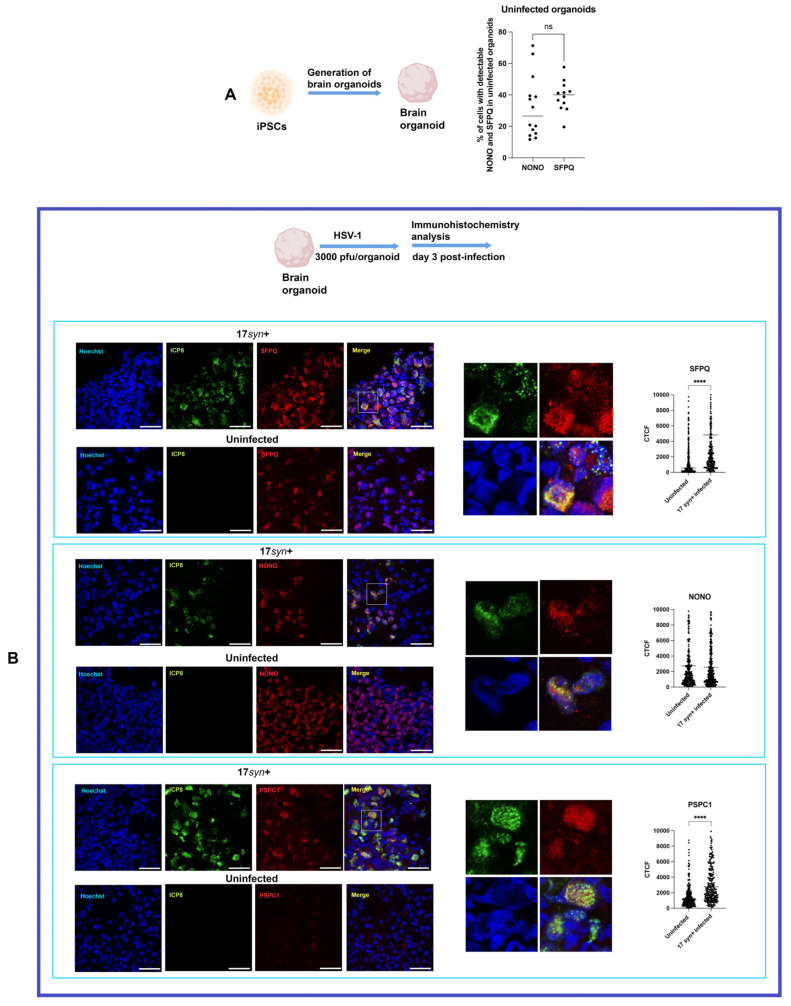
Expression and distribution of paraspeckle proteins NONO, SFPQ, and PSPC1 in uninfected and HSV-1-infected brain organoids. (**A**) Sixty-day-old cortical organoids were derived from human iPSC lines SC0000019 and SC0000020. The graph shows the distribution of cells with detectable levels of NONO and SFPQ in uninfected organoids. Each dot represents an individual microscopy field. (**B**) Cortical organoids were infected with HSV-1, strain 17*syn*^+^, (3000 pfu/organoid), and analyzed on day 3 post-infection. Representative immunofluorescence images show the subcellular localization of the paraspeckle proteins SFPQ, NONO, and PSPC1 in uninfected and infected organoids. In HSV-1-infected organoids, paraspeckle proteins exhibit marked nuclear redistribution and accumulate in Hoechst-negative nuclear regions that are positive for the viral protein ICP8, likely corresponding to HSV-1 replication compartments. The immunofluorescence signals are presented with separate channels. Higher-magnification insets highlight the spatial relationship between paraspeckle proteins and ICP8 within infected nuclei. Quantification of SFPQ, NONO, and PSPC1 fluorescence intensity in uninfected and HSV-1-infected nuclei was performed using corrected total cell fluorescence (CTCF). Quantitative analyses were conducted on three independent organoids per condition (n = 3), with multiple microscopy fields analyzed per organoid and averaged prior to statistical testing. Data are presented as the mean ± SD. Statistical significance was assessed using an unpaired Student’s t-test (**** *p* < 0.0001; ns, not significant). Nuclei were counterstained with Hoechst 33342 (blue). Scale bars, 25 μm.

The kinetics of HSV-1 replication are slower in brain organoids than in 2D neuronal cultures. This more physiological model, characterized by greater cellular heterogeneity and a transcriptomic landscape closer to native brain tissue, may lead to differences in host response to infection compared to 2D cultures, while also revealing insights not accessible in 2D cultures. Thus, we investigated the dynamics of the paraspeckle proteins NONO, SFPQ, and PSPC1 in hiPSC-derived brain organoids (Figure 3A) infected with HSV-1.

Sixty-day-old cortical organoids were infected with 3000 pfu of HSV-1 (Figure 3B) as previously described [13]. After 2 h, the inocula were removed, and organoids were analyzed on day 3 post-infection (Figure 3B). This time point was selected because our previous results show that HSV-1 infection requires about three days to penetrate to a depth of 300–350 microns within organoids [13]. The analysis of cells with detectable NONO and SFPQ revealed a heterogeneous distribution in uninfected organoids: min 11.67%, max 71.35%, mean 32.13% ± 19.74 SD, SEM 5.28; SFPQ: min 19.61%, max 57.69%, mean 39.24% ± 9.73 SD, SEM 2.08) (Figure 3A). A statistical comparison by t-test indicated no significant difference between NONO and SFPQ (Figure 3A). The observed variability in the percentages of cells expressing detectable NONO and SFPQ likely reflects the intrinsic regional heterogeneity of brain organoids.

The expression of PSPC1 in uninfected organoids was negligible (Figure 3B). In contrast, a substantial proportion of cells in monolayer neuronal cultures exhibited detectable expression of NONO and SFPQ proteins, reflecting the greater cellular diversity and region-specific differentiation inherent to 3D organoid models versus the more uniform cell populations in 2D monolayer cultures.

Immunohistochemistry analysis of HSV-1-infected organoids revealed, consistent with the observation in 2D neuronal cultures, a redistribution and accumulation of the paraspeckle proteins SFPQ, NONO, and PSPC1 within Hoechst-negative nuclear regions that were positive for ICP8, which likely correspond to VRCs (B). The analysis of the colocalization between ICP8 and NONO revealed that 55.53% of the ICP8 signal overlapped with NONO, whereas only 28.45% of the NONO signal overlapped with ICP8, indicating that while NONO accumulates specifically in the VRCs marked by ICP8, it localizes to distinct subregions within these compartments (Figure S3).

Quantification of fluorescence intensity in HSV-1-infected organoids on day 3 post-infection, measured as corrected total cell fluorescence (CTCF), showed a significant increase in SFPQ (*p* < 0.0001) but not NONO (Figure 3B), which may suggest a specific involvement of SFPQ in the antiviral response [17]. In contrast to NONO and SFPQ, only a faint immunoreactivity for PSPC1 was detectable and restricted to a small fraction of cells.

However, following HSV-1 infection a marked increase in PSPC1 immunoreactivity (*p* < 0.0001) and its accumulation in VRCs was observed (Figure 3B). The significantly higher and sustained fluorescence intensity for SFPQ and PSPC1 in infected organoids on day 3 post-infection contrasts with the transient elevation and subsequent decline in monolayer neuronal cultures, probably reflecting the complexity and the physiological relevance of these 3D culture models that better recapitulate the in vivo brain environment, where sustained viral infection and cellular responses may occur over a longer time frame. In contrast, in monolayer cultures of neurons (2D), the increase in PSPC1 and NONO is transient, peaking at 12 hpi and then declining to the baseline by 24 hpi. The stable levels of NONO could reflect distinct regulation or a more transient role during infection.

**Figure 4:**
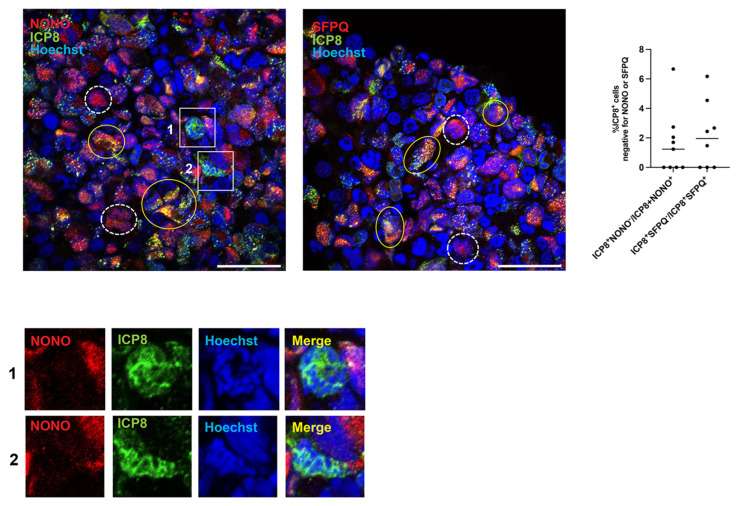
**Expression of ICP8 in relation to NONO and SFPQ in HSV-1-infected brain organoids.** Cortical organoids were generated and infected as described in Figure 3 and analyzed on day 3 p.i. Top panels. Immunohistochemistry analysis showed HSV-1 ICP8 protein expression predominantly in nuclei exhibiting detectable NONO (Top panel, left) and SFPQ (Top panel, middle) immunoreactivity in viral replication compartments. Top panel, right: Quantification of the percentage of ICP8-positive cells that lack detectable NONO or SFPQ expression, expressed relative to the total population of cells positive for either ICP8 and NONO or ICP8 and SFPQ. Each dot represents the percentage calculated from an individual microscopy field obtained from confocal images of three independent infected organoids (n = 3). Examples of cells exhibiting simultaneous detection of HSV-1 ICP8 and NONO or SFPQ immunoreactivity are indicated by green ovals. Examples of cells where ICP8 is not expressed in cells with detectable expression of NONO or SFPQ are indicated by dashed white ovals. Instances in which ICP8 is detected in cells lacking immunoreactivity for NONO are indicated by white squares and shown in the insets in the Bottom panel. Nuclei were counterstained with Hoechst 33342 (blue). Scale bars: 25 μm.

Importantly, immunohistochemical analysis revealed HSV-1 ICP8 protein expression predominantly in cells exhibiting detectable immunoreactivity for NONO and SFPQ (Figure 4). The percentage of ICP8^+^NONO^−^ cells relative to the population of ICP8^+^NONO^+^ cells in HSV-1-infected organoids ranged from 0% to 6.667%, with a mean of 1.597% (standard deviation = 2.166%). Similarly, the percentage of ICP8^+^SFPQ^−^ cells relative to the population of ICP8^+^SFPQ^+^ cells in infected organoids ranged from 0% to 6.173%, with a mean of 2.162% (standard deviation = 2.287%) (Figure 4). These results suggest that NONO and SFPQ proteins may influence nuclear architecture [18,19,20,21] to facilitate the formation of VRCs or other nuclear domains that support ICP8 function, thereby promoting efficient HSV-1 replication and viral gene expression. Thus, in our infected organoid model, we observed a heterogeneous cellular response that falls broadly into three groups: (i) Uninfected or abortively infected cells, which show no observable paraspeckle proteins; (ii) Infected cells, where ICP8 expression co-occurs with detectable levels of NONO or SFPQ; and (iii) Uninfected or abortively infected cells with detectable NONO or SFPQ but no ICP8 expression (Figure 4). In HSV-1-infected organoids, ICP8^+^ cells lacking detectable SFPQ or NONO expression were rarely observed; these cells exhibited impaired formation of VRCs (Figure 4 and Figure S4), suggesting the importance of SFPQ and NONO for the proper establishment of the viral replication compartment in permissive cells.

### 3.3. Dynamics of Paraspeckles in HSV-1-Infected Neural Precursor Cells (NPCs)

**Figure 5:**
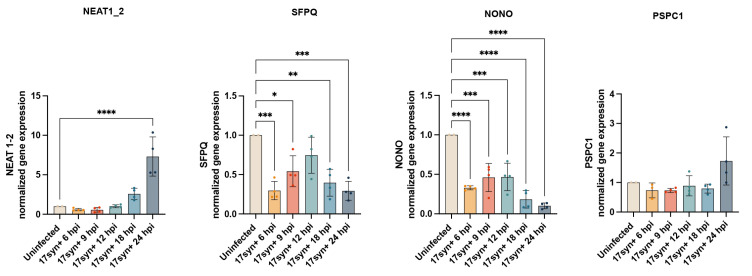
**Time-course analysis of gene expression for paraspeckle genes NEAT1_2, NONO, SFPQ, and PSPC1 in HSV-1-infected neural precursor cells.** Neural precursor cells were infected with HSV-1 and harvested at the indicated time points post-infection. Relative mRNA levels of genes NEAT1_2, NONO, SFPQ, and PSPC1 were quantified by RT-qPCR. Transcript levels were normalized to HPRT and expressed relative to uninfected control cells. Error bars represent standard deviations. Statistical comparisons between uninfected and infected conditions at each time point were performed using one-way analysis of variance (ANOVA), followed by Tukey’s multiple-comparison post hoc test. Statistical significance is indicated as follows: * *p* = 0.02; ** *p* = 0.002; *** *p* < 0.001; **** *p* < 0.0001.

Given the observed divergence in the temporal expression of paraspeckle genes in HSV-1-infected post-mitotic neurons compared to previously published results in proliferating HeLa cells and mouse embryonic fibroblasts (MEFs), we conducted a time-course analysis of the expression of paraspeckle genes in infected neural precursor cells (NPCs). NPCs were chosen for their proliferative nature, which is analogous to that of HeLa cells and MEFs. NPCs were derived from hiPSCs as previously described [15] and infected with HSV-1 at an MOI of 0.1 (Figure 5).

The expression of the paraspeckle-associated genes was analyzed at multiple time points (6–24 h post-infection). RT-qPCR analysis showed increased expression of NEAT1_2 starting at 18 hpi (though not statistically significant), with significant upregulation at 24 hpi (*p* < 0.0001). The analysis of NONO gene expression in infected NPC cultures revealed a progressive decrease across the different time points examined, indicating a more rapid response when compared to neurons, where a significant reduction in the expression of this gene was detected starting from 18 hpi (Figure 5). SFPQ expression was differentially regulated between NPCs and neurons. Specifically, SFPQ expression showed a significant decrease at 6 hpi (*p* = 0.0007), which was followed by a progressive increase at 9 hpi (*p* = 0.0206) and 12 hpi, with the expression level at 12 hpi being comparable to that of uninfected cultures. Subsequently, SFPQ expression decreased again significantly at 18 hpi and 24 hpi (*p* = 0.0027; *p* = 0.0006) when compared to uninfected cultures (Figure 5). This temporal expression profile of SFPQ is distinct from that observed in neuronal cells, where a significant upregulation was detected as early as 6 hpi and remained significantly elevated throughout all analyzed time points (Figure 5). No significant changes in PSPC1 expression were observed in infected NPCs at any of the time points analyzed, in contrast to infected neurons, where a significant upregulation was observed following infection, peaking at 9 hpi (Figure 5).

These findings highlight differences in host response to HSV-1 among different cell types and during different stages of neuronal differentiation and/or could reflect different strategies employed by the virus to utilize host pathways in different cellular environments.

## 4. Discussion

Recent reports have highlighted the critical role of paraspeckles as key regulators of host–virus interactions by modulating viral infections through diverse mechanisms, including the activation of antiviral gene expression, fine-tuning of interferon responses, and regulation of viral gene expression, in non-neuronal cells [3,8,9,11,22,23,24,25]. To our knowledge, no studies have investigated the dynamics of paraspeckle components in HSV-1-infected neuronal cells, which represent a unique cellular environment, characterized by highly specialized transcriptional programs and complex nuclear architecture [26,27].

In this study, we investigated the changes of expression of paraspeckle-associated genes in hiPSC-derived monolayer cultures of NPCs and neurons and in 3D brain cortical organoids following HSV-1 infection.

First, our results show cell-type-specific variations in host responses to HSV-1 infection and likely reflect distinct strategies employed by the virus to exploit host pathways in different cellular environments.

In HeLa cells and mouse embryonic fibroblasts (MEFs) [11], HSV-1 infection rapidly activates STAT3, which upregulates NEAT1_2 expression. The nuclear speckle protein SRSF2 interacts with paraspeckle components, including NEAT1, PSPC1, and NONO, all of which bind HSV-1 genomic DNA. PSPC1 mediates recruitment of STAT3 to paraspeckles, facilitating STAT3 binding to viral promoters, such as ICP0 and thymidine kinase (TK), thereby enhancing viral gene transcription [11]. NEAT1_2 in HSV-1-infected HeLa and MEFs is upregulated during the early stage of the infection, peaking at 4 hpi, followed by a decline. The expression of the paraspeckle proteins NONO and PSPC1 remain unchanged at all considered time points in HSV-1 HeLa cells [11]. In contrast, our results reveal a distinct expression pattern during lytic HSV-1 infection in hiPSC-derived cortical neurons, where (i) NEAT1_2 showed an increase over time during the infection of neurons, with a statistically significant increase at 18 hpi (Figure 1B), and (ii) the expression of the proteins NONO, SFPQ, and PSPC1 showed a biphasic regulation during HSV-1 infection of neurons, where the expression of these proteins increased progressively, peaking at 12 hpi, followed by a decline to levels comparable to those in uninfected cells (Figure 2). This may indicate that HSV-1 exploits specific paraspeckle components transiently, then triggers their degradation to prevent additional antiviral responses.

Immunocytochemistry analysis revealed a redistribution of the paraspeckle proteins SFPQ, NONO and PSPC1 and their accumulation in the VRC, a phenomenon never reported in HSV-1-infected non-neuronal cells, which may represent another cell-type-specific response that underlies both host defense mechanisms or the ability of HSV-1 to exploit neuronal machinery.

At the transcriptional level, the expression of PSPC1 and SFPQ increased significantly in infected neurons starting from 6 hpi and subsequently declined to baseline levels at 18 hpi and 24 hpi, respectively. In contrast, NONO expression remained unchanged until 12 hpi, after which levels decreased significantly to the baseline at 18 hpi. Together, these results reinforce the notion that the activities of NONO, SFPQ, and PSPC1 are mainly required during early stages of the infection.

Collectively, the analysis of the expression of the paraspeckle components and the dynamics of the redistribution of the paraspeckle proteins in HSV-1-infected neurons at different time points, together with the observed upregulation of NEAT1_2 RNA at 24 hpi, when the abundance of the aforementioned paraspeckle protein declines, poses the question of whether the paraspeckle proteins accumulating in the VRCs localize within paraspeckles, remain as free proteins in the nucleoplasm, or are incorporated into different nuclear aggregates.

Considering the differences we observed in paraspeckle gene expression in HSV-1-infected neurons compared with the patterns previously reported in proliferating cells such as HeLa cells and MEFs, we next investigated whether the temporal expression patterns of paraspeckle-associated genes are comparable between proliferating neuronal cells (NPCs) and non-proliferating mature neurons.

At 24 h post-infection, the regulatory trends of paraspeckle genes were comparable in neurons and NPCs, indicating similar directions of expression change (NONO was down-regulated, NEAT1_2 was upregulated, and PSPC1 was comparable to uninfected cells) despite differences in magnitude. However, the temporal changes during earlier stages differ markedly between the two cell types. Also, SFPQ expression exhibited opposing regulatory patterns between NPCs and neurons. Considering that SFPQ acts as a negative regulator of HSV-1 gene expression (knockdown of SFPQ in HSV-1-infected cells leads to increased transcription of viral enhancements in HSV-1 gene expression [11]), its down-regulation in HSV-1-infected NPCs at all the time points analyzed may represent a mechanism contributing to the higher permissiveness of NPCs compared to neurons to HSV-1.

The analysis of brain organoids revealed important insights into the expression patterns of NONO and SFPQ and their relationship with ICP8 expression. Immunohistochemistry analysis of uninfected brain organoids revealed a more heterogeneous distribution of cells with detectable expression of NONO and SFPQ compared to monolayer neuronal cultures, in which a larger and more uniform proportion of cells expressed both proteins. In infected organoids, HSV-1 ICP8 expression was mainly detected in cells with detectable levels of NONO and SFPQ. Thus, in infected organoids, the population of cells with detectable NONO or SFPQ fell into two groups: (i) infected cells (ICP8-positive) and (ii) uninfected or abortively infected cells (ICP8-negative). Only a negligible fraction of ICP8-positive cells was found to lack immunoreactivity for SFPQ and NONO; however, in these cells no Hoechst-negative nuclear regions coinciding with the ICP8 signal were detected, indicating that the formation of VRCs was impaired. These results indicate that NONO and SFPQ expressions are associated with HSV-1 permissiveness. HSV-1 may productively infect cells with detectable SFPQ and NONO because these paraspeckle proteins are involved in various aspects of RNA processing and nuclear architecture [28,29]. It is plausible that these proteins are required to establish or maintain the nuclear environment that supports the assembly of HSV-1 replication compartments; however, directly demonstrating this in our experimental system will require further structural and functional investigation.

Together, these results lead us to hypothesize a dual role of SFPQ and NONO in HSV-1 infection, as both are initially required for permissiveness to HSV-1, but their functions diverge during the infection (according to the current literature), becoming more anti- and proviral factors, respectively. This may provide yet another example of a virus evolving to use and subvert cellular defenses to promote infection programs which may vary in different cell types.

A limitation of the present study is the absence of loss- and gain-of-function experiments to formally demonstrate the functional roles of NONO, SFPQ, and PSPC1 in HSV-1 infection of neuronal cells. Future studies employing targeted silencing of these paraspeckle proteins will be important to establish their precise functional contribution to HSV-1 replication.

## 5. Conclusions

Our study provides the first evidence of cell-type-specific differences in the expression of paraspeckle-associated genes during HSV-1 infection in neurons, alongside a redistribution of paraspeckle proteins into VRCs. Notably, the expression of the paraspeckle proteins NONO and SFPQ associate with HSV-1 permissiveness in human neuronal cells, suggesting these proteins contribute to creating a nuclear environment conducive to viral transcription and replication.

## Data Availability

The data supporting the findings of this study (raw RT-qPCR data, original Western blot images, and immunohistochemistry microscopy images) are available from the corresponding author upon reasonable request.

## Supplementary Materials

The following supporting information can be downloaded at: https://www.mdpi.com/article/10.3390/v18050552/s1, Figure S1: Co-localization analysis between HSV-1 ICP8 and paraspeckle proteins NONO, SFPQ, and PSPC1; Figure S2: Full-length western blot images; Figure S3: Colocalization analysis of NONO and ICP8 in HSV-1 infected organoids; Figure S4: Immunohistochemistry analysis of HSV-1 infected 60-day old brain organoid depicting the expression of HSV-1 ICP8 (green) in cells with detectable NONO (A, red) or SFPQ (B, red).

## Abbreviations

The following abbreviations are used in this manuscript:

HiPSCs	Human induced pluripotent stem cells
NPCs	Neural precursor cells
HSV-1	Herpes Simplex Virus 1

## References

[1] Wang Y., Chen L.L. (2020). Organization and function of paraspeckles. Essays Biochem..

[2] Hirose T., Virnicchi G., Tanigawa A., Naganuma T., Li R., Kimura H., Yokoi T., Nakagawa S., Bénard M., Fox A.H. (2014). NEAT1 long noncoding RNA regulates transcription via protein sequestration within subnuclear bodies. Mol. Biol. Cell.

[3] Li K., Wang Z. (2021). Speckles and paraspeckles coordinate to regulate HSV-1 genes transcription. Commun. Biol..

[4] Bond C.S., Fox A.H. (2009). Paraspeckles: Nuclear bodies built on long noncoding RNA. J. Cell Biol..

[5] Fox A.H., Lamond A.I. (2010). Paraspeckles. Cold Spring Harb. Perspect. Biol..

[6] Cardinale S., Cisterna B., Bonetti P., Aringhieri C., Biggiogera M., Barabino S.M. (2007). Subnuclear localization and dynamics of the Pre-mRNA 3′ end processing factor mammalian cleavage factor I 68-kDa subunit. Mol. Biol. Cell..

[7] Grosch M., Ittermann S., Rusha E., Greisle T., Ori C., Truong D.-J.J., O’neill A.C., Pertek A., Westmeyer G.G., Drukker M. (2020). Nucleus size and DNA accessibility are linked to the regulation of paraspeckle formation in cellular differentiation. BMC Biol..

[8] Milcamps R., Michiels T. (2024). Involvement of paraspeckle components in viral infections. Nucleus.

[9] Harper K.L., Harrington E.M., Hayward C., Anene C.A., Wongwiwat W., White R.E., Whitehouse A. (2024). Virus-modified paraspeckle-like condensates are hubs for viral RNA processing and their formation drives genomic instability. Nat. Commun..

[10] Lee N., Yario T.A., Gao J.S., Steitz J.A. (2016). EBV noncoding RNA EBER2 interacts with host RNA-binding proteins to regulate viral gene expression. Proc. Natl. Acad. Sci. USA.

[11] Wang Z., Fan P., Zhao Y., Zhang S., Lu J., Xie W., Jiang Y., Lei F., Xu N., Zhang Y. (2017). NEAT1 modulates herpes simplex virus-1 replication by regulating viral gene transcription. Cell Mol. Life Sci..

[12] Cagno V., Civra A., Rossin D., Calfapietra S., Caccia C., Leoni V., Dorma N., Biasi F., Poli G., Lembo D. (2017). Inhibition of herpes simplex-1 virus replication by 25-hydroxycholesterol and 27-hydroxycholesterol. Redox Biol..

[13] Abrahamson E.E., Zheng W., Muralidaran V., Ikonomovic M.D., Bloom D.C., Nimgaonkar V.L., D’Aiuto L. (2020). Modeling Aβ42 Accumulation in Response to Herpes Simplex Virus 1 Infection: 2D or 3D?. J. Virol..

[14] Schindelin J., Arganda-Carreras I., Frise E., Kaynig V., Longair M., Pietzsch T., Preibisch S., Rueden C., Saalfeld S., Schmid B. (2012). Fiji: An open-source platform for biological-image analysis. Nat. Methods.

[15] D’Aiuto L., Caldwell J.K., Edwards T.G., Zhou C., McDonald M.L., Di Maio R., Joel W.A., Hyde V.R., Wallace C.T., Watkins S.C. (2025). Phosphorylated-tau associates with HSV-1 chromatin and correlates with nuclear speckles decondensation in low-density host chromatin regions. Neurobiol. Dis..

[16] Naganuma T., Hirose T. (2013). Paraspeckle formation during the biogenesis of long non-coding RNAs. RNA Biol..

[17] Imamura K., Imamachi N., Akizuki G., Kumakura M., Kawaguchi A., Nagata K., Kato A., Kawaguchi Y., Sato H., Yoneda M. (2014). Long Noncoding RNA NEAT1-Dependent SFPQ Relocation from Promoter Region to Paraspeckle Mediates IL8 Expression upon Immune Stimuli. Mol. Cell.

[18] Petti E., Buemi V., Zappone A., Schillaci O., Broccia P.V., Dinami R., Matteoni S., Benetti R., Schoeftner S. (2019). SFPQ and NONO suppress RNA:DNA-hybrid-related telomere instability. Nat. Commun..

[19] Sas-Nowosielska H., Magalska A. (2021). Long Noncoding RNAs-Crucial Players Organizing the Landscape of the Neuronal Nucleus. Int. J. Mol. Sci..

[20] Kawaguchi T., Tanigawa A., Naganuma T., Ohkawa Y., Souquere S., Pierron G., Hirose T. (2015). SWI/SNF chromatin-remodeling complexes function in noncoding RNA-dependent assembly of nuclear bodies. Proc. Natl. Acad. Sci. USA.

[21] Kathman S.G., Koo S.J., Lindsey G.L., Her H.-L., Blue S.M., Li H., Jaensch S., Remsberg J.R., Ahn K., Yeo G.W. (2023). Remodeling oncogenic transcriptomes by small molecules targeting NONO. Nat. Chem. Biol..

[22] Payen S.H., Andrada K., Tara E., Petereit J., Verma S.C., Rossetto C.C. (2024). The cellular paraspeckle component SFPQ associates with the viral processivity factor ORF59 during lytic replication of Kaposi’s Sarcoma-associated herpesvirus (KSHV). Virus Res..

[23] Friedl M.S., Djakovic L., Kluge M., Hennig T., Whisnant A.W., Backes S., Dölken L., Friedel C.C. (2022). HSV-1 and influenza infection induce linear and circular splicing of the long NEAT1 isoform. PLoS ONE.

[24] Beeharry Y., Goodrum G., Imperiale C.J., Pelchat M. (2018). The Hepatitis Delta Virus accumulation requires paraspeckle components and affects NEAT1 level and PSP1 localization. Sci. Rep..

[25] Cao S., Moss W., O’Grady T., Concha M., Strong M.J., Wang X., Yu Y., Baddoo M., Zhang K., Fewell C. (2015). New Noncoding Lytic Transcripts Derived from the Epstein-Barr Virus Latency Origin of Replication, oriP, Are Hyperedited, Bind the Paraspeckle Protein, NONO/p54nrb, and Support Viral Lytic Transcription. J. Virol..

[26] Baumann N., Morassut I., Roig-Puiggros S., Klingler E., Bartolini G., Fièvre S., Jabaudon D. (2025). Cell-extrinsic controls over neocortical neuron fate and diversity. Sci. Adv..

[27] Ito K., Takizawa T. (2018). Nuclear Architecture in the Nervous System: Development, Function, and Neurodevelopmental Diseases. Front. Genet..

[28] Zhang S., Cooper J.A.L., Chong Y.S., Naveed A., Mayoh C., Jayatilleke N., Liu T., Amos S., Kobelke S., Marshall A.C. (2023). NONO enhances mRNA processing of super-enhancer-associated GATA2 and HAND2 genes in neuroblastoma. EMBO Rep..

[29] Koning H.J., Lai J.Y., Marshall A.C., Stroeher E., Monahan G., Pullakhandam A., Knott G.J., Ryan T.M., Fox A.H., Whitten A. (2025). Structural plasticity of the coiled-coil interactions in human SFPQ. Nucleic Acids Res..

