# Supplementary figures and images for: Dynamics of Paraspeckle Components in Herpes Simplex Virus 1 (HSV-1)-Infected Human Neuronal Cells
Source: Viruses. 2026 May 12;18(5):552. doi: 10.3390/v18050552 (PMC13211404; doi:10.3390/v18050552)

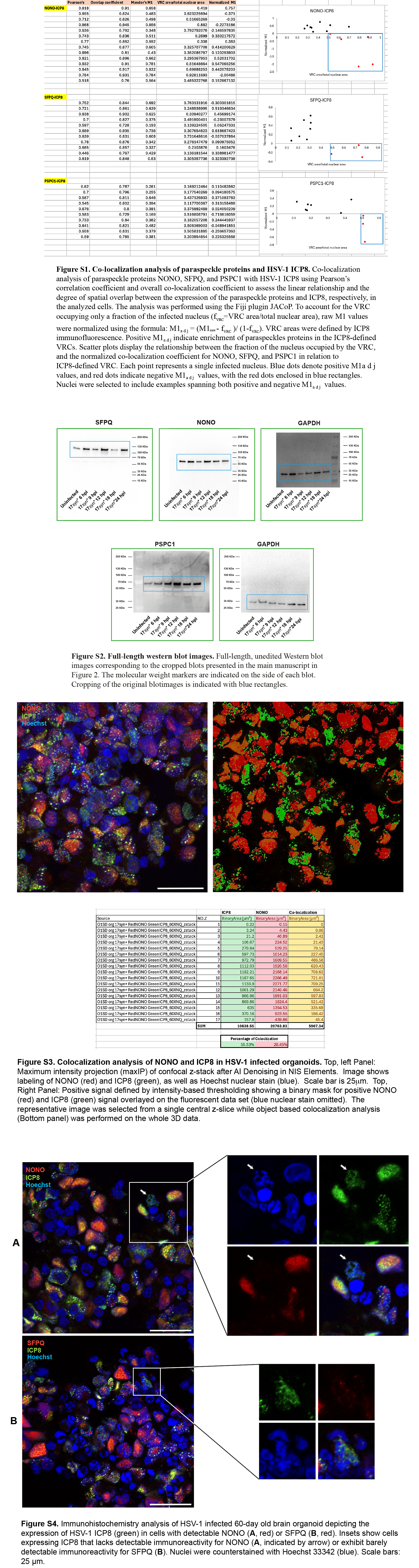

Supplement: Supplementary file 1 [file viruses-18-00552-s001.zip › viruses-4250733-supplementary.tif]
